# Brain-to-blood transport of fluorescein in vitro

**DOI:** 10.1038/s41598-024-77040-2

**Published:** 2024-10-26

**Authors:** Karl Schoknecht, Jens Eilers

**Affiliations:** Carl-Ludwig-Institute of Physiology, Medical Faculty, Leipzig University, Liebigstr. 27, 04103 Leipzig, Germany

**Keywords:** Blood-brain barrier, Fluorescein, Organic anion transporting polypeptide, Efflux, Oxygen-glucose deprivation, Blood-brain barrier, Transporters in the nervous system

## Abstract

Investigating blood-brain barrier (BBB) dysfunction has become a pre-clinical and clinical research focus as it accompanies many neurological disorders. Nevertheless, knowledge of how diagnostic BBB tracers cross the endothelium from blood-to-brain or vice versa often remains incomplete. In particular, brain-to-blood transport (efflux) may reduce tracer extravasation of intravascularly (i.v.) applied tracers. Conversely, impaired efflux could mimic phenotypic extravasation. Both processes would affect conclusions on BBB properties primarily attributed to blood-to-brain leakage. Here, we specifically investigated efflux of fluorescent BBB tracers, focusing on the most common non-toxic marker, sodium fluorescein, which is applicable in patients. We used acute neocortical slices from mice and applied fluorescein, sulforhodamine-B, rhodamine-123, FITC dextran to the artificial cerebrospinal fluid. Anionic low molecular weight (MW) fluorescein and sulforhodamine-B, but not ~ 10-fold larger FITC-dextran and cationic low MW rhodamine-123, showed efflux into the lumen of blood vessels. Our data suggest that fluorescein efflux depends on organic anion transporter polypeptides (Oatp) rather than P-glycoprotein. Furthermore, sodium-potassium ATPase inhibition and incomplete oxygen-glucose deprivation (OGD, 20% O_2_) reduced fluorescein efflux, while complete OGD (0% O_2_) abolished efflux. We provide evidence for active efflux of fluorescein in vitro. Impaired efflux of fluorescein could thus contribute to the frequently observed BBB dysfunction in neuropathologies in addition to blood-to-brain leakage.

**Supplementary Information:**

The online version contains supplementary material available at 10.1038/s41598-024-77040-2.

## Introduction

The blood-brain barrier (BBB) comprises several structural and functional features of the cerebral endothelium that are essential for regulating extracellular fluid composition and nourishment of the central nervous system (CNS). Compared with peripheral blood vessels, CNS blood vessels conduct less vesicular transcytosis and less paracellular transport as tight junction proteins seal the paracellular space^1,2^. In addition, cerebral endothelial cells express a variety of transport proteins (e.g. ATP-binding cassette (ABC) transporter) that remove substances from the *brain* to the *blood*, and solute carriers including organic anion transporting polypeptides (Oatp) that can act as transporters from *blood* to *brain *or vice versa^2,3^. While endothelial cells ultimately mediate transport across the BBB, astrocytes, pericytes, microglia and neurons are supportive cell types that play a regulatory role^4–7^.

BBB dysfunction, usually referring to increased BBB permeability, has been found in various neurological disorders, including epilepsy, brain tumors, multiple sclerosis, ischemic and hemorrhagic stroke, traumatic brain injury, mild cognitive impairment, and dementia^8–13^. A causative pathophysiological role has been attributed to BBB dysfunction in some of these disorders, e.g. for hemorrhagic transformation or cerebral edema following ischemic stroke^14,15^, and for epileptogenesis^10,16–18^. Apart from pathological alterations, BBB permeability has been shown to undergo diurnal changes in mice and was increased following extensive sensory stimulation in rats and motor exercise in humans^19–21^. Interestingly, blocking the increase in BBB permeability following sensory stimulation reduced the otherwise potentiated sensory evoked potentials^21^. Thus, the BBB has evolved as an element in understanding neurological disorders, and, more recently, physiological neuronal plasticity.

Current epidemiological knowledge on BBB dysfunction in diseases emerged primarily from imaging studies. These studies typically rely on intravascularly (i.v.)-injected tracer molecules that are largely impermeable to a healthy BBB, followed by their detection in the extravascular (e.v.) compartment in vivo, in vitro or after tissue fixation^22–25^. Notably, i.v.-application of tracers is inherently more suitable to detect alterations in *blood* to *brain* rather than *brain* to *blood *transport, yet extravascular tracer signals, e.g. fluorescence intensity, could be affected by both modes of transport across the BBB. Among the low toxicity markers, fluorescein has been used most frequently (certainly by number of publications), predominantly in animal experiments but also to assess blood-retinal barrier permeability and borders of brain tumors in humans^25–29^. Although fluorescein was introduced to BBB research around 1960^30^, the pathway across the BBB, whether from *blood* to *brain* or from *brain* to *blood *after extravasation, remain largely unknown^25^. One in vivo study demonstrated an increased brain-plasma ratio of i.v.-injected fluorescein when probenecid, an inhibitor of Oatp, was co-infused with fluorescein in rats^31^. Therefore, this increased fluorescein content in the brain, could be explained by reduced transport from *brain* to *blood*, hereafter referred to as ‘efflux’. This is supported by data showing reduced brain fluorescein content when multidrug-resistance associated protein-2 (Mrp2) expression was upregulated^32^. Taken together, these studies suggest active efflux of fluorescein, although luminal accumulation of fluorescein has not been shown directly.

Here, we performed imaging experiments to test the hypothesis that fluorescein undergoes efflux and to explore underlying mechanisms in acute brain slices from adult mice. We indeed show efflux of fluorescein, presumably by Oatp. Our findings complement conclusions from imaging studies using fluorescein as a marker to measure BBB permeability. In addition to *blood* to *brain* ‘leakage’, fluorescein extravasation following i.v.-injection may be sensitive to changes in efflux.

## Results

### Fluorescein accumulates in cerebral blood vessels in vitro

**Fig. 1:**
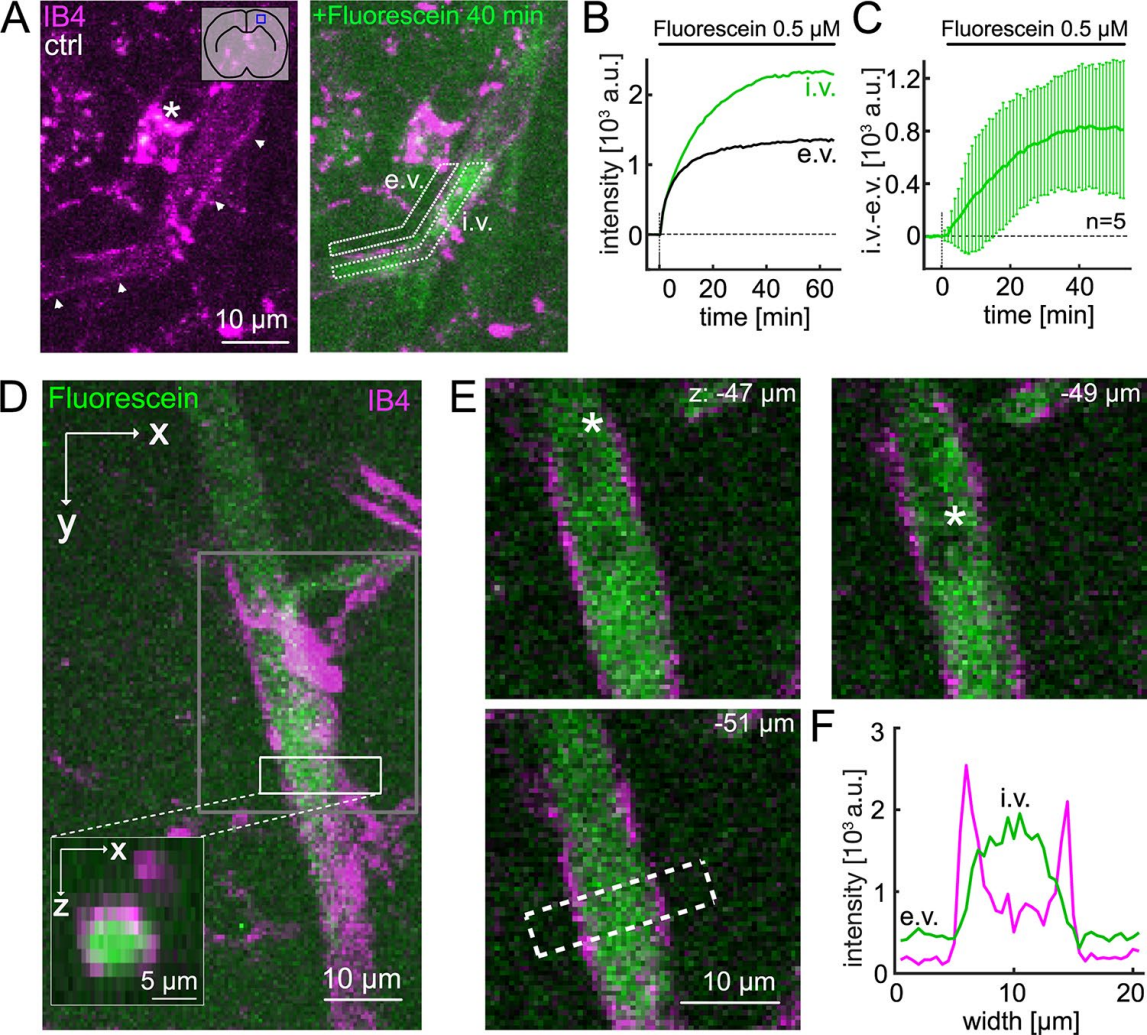
Fluorescein accumulates in cerebral blood vessels in vitro. (**A**) Two color z-projection images (17 z-planes taken at 1.5 μm intervals, 30–54 μm below the slice surface, maximum intensity projection) of a vessel fragment (arrowheads) in the somatosensory cortex of mouse brain slice stained by IB4 (magenta, 1.45 nM, endothelial cell and microglial (*) marker, applied for 1 h after slicing) without prior fluorescein labeling (ctrl, left) and following 40 min of wash-in of fluorescein (0.5 µM; green, right). The blue square in pictogram (left panel, top right corner) illustrates approximate position of the image in the brain slice. Dotted lines surrounding ‘e.v.’ (extravascular) and ‘i.v.’ (intravascular) denote ROIs used for quantification of the average signal during wash-in of fluorescein shown in B. (**B**) Corresponding e.v. (black) and i.v. (green) fluorescein intensity curves during wash-in of fluorescein (0.5 µM). Control values were subtracted. Data were acquired at 1 min^-1^. Note the intraluminal accumulation of fluorescein. (**C**) Summary of wash-in experiments (*n* = 5 slices, 4 mice) presented as the mean difference between i.v. and e.v. fluorescein intensity (whiskers extend to ± one standard deviation). (**D**) Another two-color z-projection of a neocortical vessel stained with fluorescein (green, 0.5 µM continuously present in the bath) and IB4. Gray box shows ROI of individual z-planes displayed in D. White box within gray box illustrates vessel fragment used for y-projection (inset, bottom left corner). Note the predominant intravascular signal of fluorescein as indicated by the surrounding endothelial layer (IB4 signal). (**E**) Three z-planes of the ROI outlined in A (gray box). Numbers in top right corner indicate the z-position relative to the slice surface. Asterisks denote putative red blood cells, which are surrounded by fluorescein and endothelial staining. The dotted white box outlines the vessel segment used for the intensity profiles in C. (**F**) Vascular profile of fluorescein and IB4 signal intensity (green and magenta, respectively). Note the substantially higher fluorescein intensity in the vascular lumen compared to the parenchyma (approximately > 5 and < 18 μm on the length scale).

**Fig. 2:**
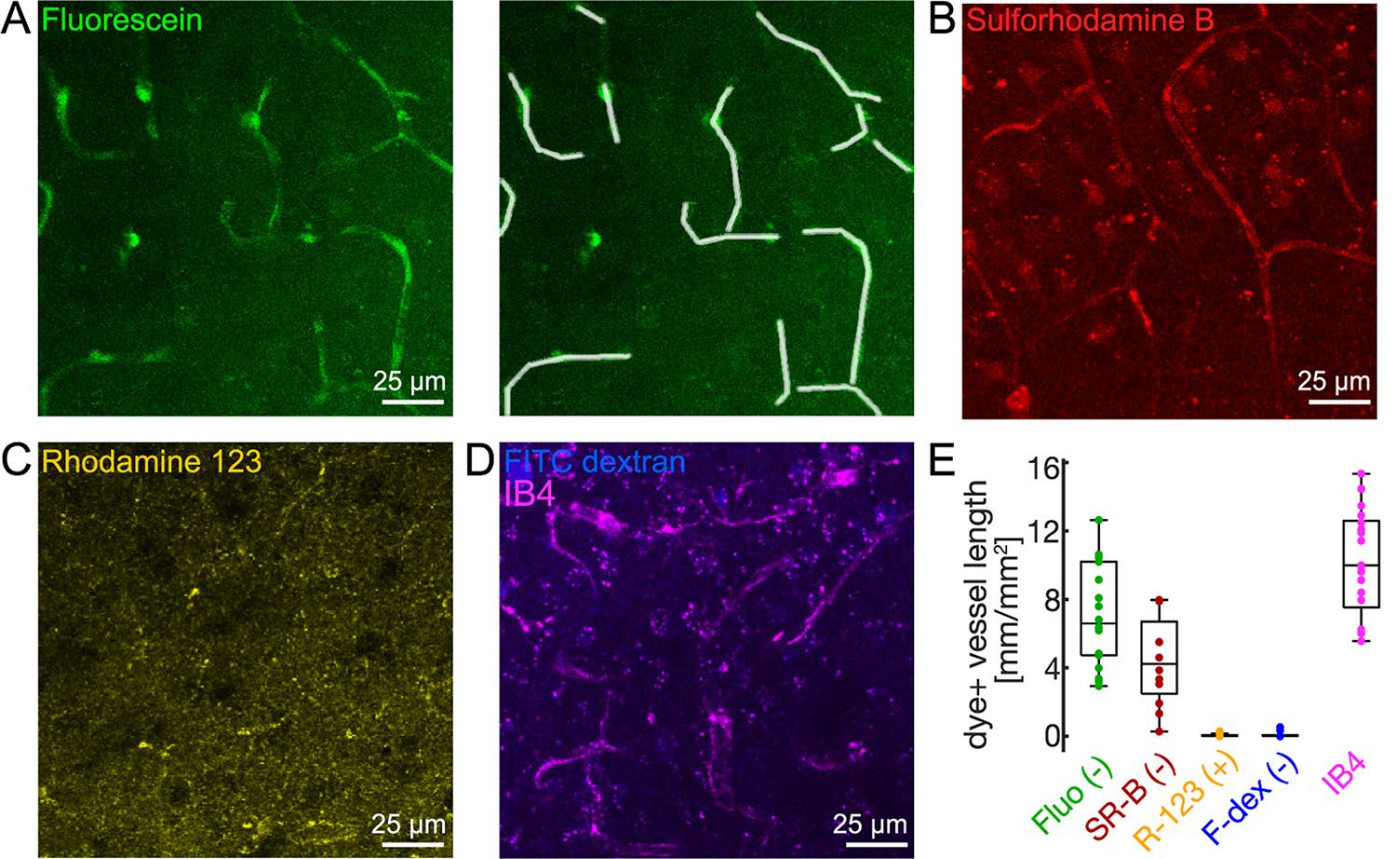
Efflux of low molecular weight anionic dyes in vitro. (**A**) Z-projection of an acute brain slice stained with fluorescein (0.5 µM, green, left; anionic, MW 332 Da). Image on the right shows the same ROI with manually traced vascular fragments (white lines) used for quantification (cf. E). (**B**-**D**) Z-projection as in A of different slices stained with sulforhodamine B (B; 0.1 µM; anionic, MW 559 Da), rhodamine 123 (C; 0.1 µM, cationic, MW 344 Da), and FITC-conjugated dextran (D, 1 µM, anionic, MW 3.000 Da) together with IB4 (1.45 nM). (**E**) Summary boxplot of dye-positive vascular fragments expressed as length per imaged z-projection area (mm/mm^2^). Three visual fields per slice were analyzed and averaged so that each slice is represented by one value in this plot. Note, that blood vessels accumulated both low MW anionic dyes, fluorescein (“Fluo (-), n = 18 slices, 7 mice) and sulforhodamine B (“SR-B (-)”, n = 12 slices, 4 mice), but not cationic low MW rhodamine 123 (“R-123 (+)”, n = 8 slices, 4 mice) or anionic FITC-dextran weighing 3000 Da (“F-dex (-)”, n = 9 slices, 3 mice). In situ IB4 staining was used to label blood vessels in a subset of slices (n = 21 slices, 5 mice).

We investigated whether fluorescein, a BBB tracer often applied i.v.^25,28,33^, is subject to efflux, i.e. is exported from the *brain parenchyma* to *blood vessels in vitro*. Cerebral blood flow (CBF) and shear-stress are absent in acute brain slices. Although this is a limitation of the slice model, it facilitates detection of tracers in blood vessels by excluding their dilution in blood as well as renal and hepatic clearance. We applied fluorescein to the e.v. compartment, thereby bypassing the BBB from blood to brain and thus mimicking extravasation. Subsequently, fluorescein accumulated in cerebral blood vessels (Fig. 1). Several aspects of this accumulation argue for actively established endothelial and luminal colocalization over mere diffusion of fluorescein into the vessel driven by a concentration gradient and/or adherence of fluorescein to blood vessels. First, monitoring of fluorescein wash-in in dye-naïve slices (Fig. 1A, left panel) for ~ 1 h showed vascular fluorescein accumulation, i.e. the establishment of a concentration gradient i.v.> e.v., reaching a plateau after ~ 30–40 min (Fig. 1A, (right panel), B,C). Second, the endothelial marker IB4 outlined a fluorescein-positive band (Fig. 1A,D-E). In slices pre-incubated with fluorescein (Fig. 1D-F), the vascular fluorescein intensity exceeded the intensity of neighboring e.v. parenchyma by factor 1.6 (1.5, 1.8; median, Q1 and Q3, respectively; *n* = 38 drug-naïve control slices from 13 mice). It is important to note that in slices pre-incubated with fluorescein or other dyes (Fig. 2), dyes were present in the artificial cerebrospinal fluid (aCSF) not only during the incubation period but also during image acquisition. Thus, increased intravascular fluorescence compared to the e.v. compartment, indicates actively established concentration gradients. Third, fluorescein signal tightly surrounded luminal erythrocytes but was absent where they were located (Fig. 1E); and fourth, fluorescein increased at the vessel wall (i.e. co-localized with IB4-positive pixels) but reached its maximum in the lumen, as shown for a single z-plane (Fig. 1E, bottom panel and Fig. 1F). Furthermore, fluorescein efflux, i.e. vascular accumulation, was absent in energy-deprived slices (cf. Figure 4). Fluorescein efflux was not selective for arterioles, capillaries or venules. Taken together, we provide evidence for a luminal localization of fluorescein as a consequence of efflux rather than diffusion.

**Fig. 3:**
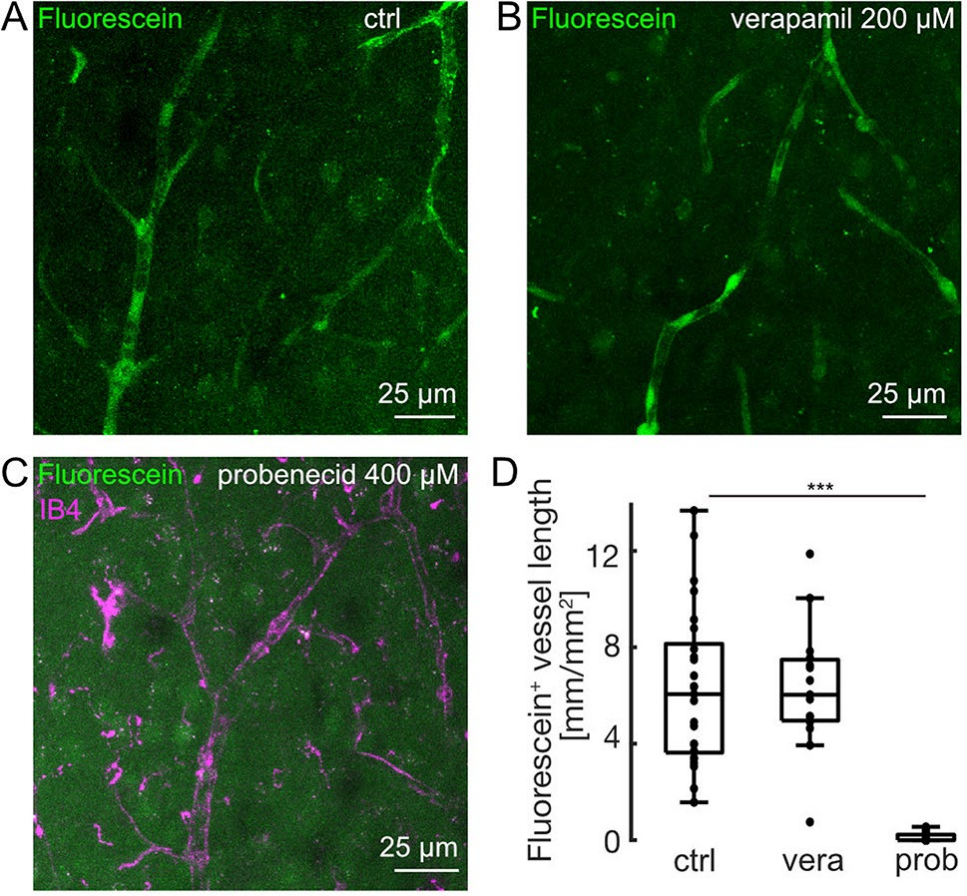
Pharmacological inhibition of efflux of fluorescein. (**A**) Z-projection of acute brain slice stained with fluorescein (0.5 µM; ctrl). (**B**) Z-projection as in A for a different slice stained with fluorescein (0.5 µM) in presence of verapamil (200 µM, inhibitor of P-glycoprotein and multidrug resistance-associated proteins). (**C**) Z-projection as in A and B for a different slice co-stained with fluorescein (0.5 µM) and IB4 (1.45 nM) in presence of probenecid (400 µM, inhibitor of organic anion transporting polypeptides and multidrug resistance-associated proteins). Note, that probenecid prevented efflux of fluorescein. (**D**) Boxplot summarizing the effect of verapamil (“vera”, *n* = 15 slices from 5 mice) and probenecid (“prob”, *n* = 16 slices from 6 mice) on the length of fluorescein-positive vessels per visual field compared to control (*n* = 25 slices from 9 mice, Mann-Whitney U test & Bonferroni post-hoc correction). Drug-treated slices were compared to independent, untreated control slices.

### Efflux of fluorescent markers differs with MW and charge

Next, we tested efflux of other fluorescent dyes that have been used as i.v. administered BBB markers, namely sulforhodamine B^34^, rhodamine 123^35^, and FITC-conjugated dextran^28^. Already qualitatively, these stains differed from fluorescein, which distinguished the vessels from the e.v. compartment with largely homogenous but lower fluorescence intensity (Fig. 2A). Sulforhodamine B similarly stained vessels and some parenchymal cells (Fig. 2B). Rhodamine 123 created clusters (Fig. 2C), presumably labeling mitochondria^36^, but did not stain blood vessels. Likewise, FITC-conjugated dextran (3000 Da) did not appear in the vascular lumen, but stained some parenchymal cells, confirming diffusion to the imaging planes (Fig. 2D).

For quantitative comparison, we measured the length of dye-positive vascular fragments by tracing them in each region of interest (ROI), i.e. in z-projections (Fig. 2A, right panel). This approach was independent of endothelial co-staining. Considering the reported intercapillary distance of 30–40 μm in the neocortex^37–39^, we could exclude that ROIs (see methods) were free of blood vessels. Nevertheless, in a subset of slices stained with IB4 we confirmed the presence of vessels in each ROI, which had a length of 10.0 mm/mm^2^ (7.5, 12.6) (*n* = 21 slices, 62 ROIs, 5 mice, Fig. 2E). Due to their abundance, capillaries were the predominant vessel subtype used for analysis, although fluorescein efflux was not selective for capillaries and also observed in arterioles. We found fluorescein-stained vessels with a length of 6.6 mm/mm^2^ (4.7, 10.2) in z-projections (Fig. 2E, *n* = 18 slices from 6 mice). Sulforhodamine B, as fluorescein an anionic low MW marker (559 Da), similarly stained vessels with a length of 4.2 mm/mm^2^ (2.5, 6.7; Fig. 2B and E, *n* = 12 slices from 4 mice) and the data distribution largely overlapped with that of fluorescein (Fig. 2E). Conversely, rhodamine 123, a low MW (344 Da) but cationic marker, or larger anionic FITC-conjugated dextran (3000 Da) did not accumulate in vessels (Fig. 2C-E, dye-positive vessels length of 0 mm/mm^2^ (0, 0.1) for both, *n* = 8 and 9 slices from 4 to 3 mice, respectively). Taken together, these data show that a subset of fluorescent indicators used to detect *blood* to *brain* leakage, here anionic low MW markers, also undergo efflux, but larger anionic and cationic markers in this cohort may not.

### Pharmacological inhibition of fluorescein efflux

Given that efflux appeared to be specific rather than universal, we wanted to determine the underlying transport mechanisms of fluorescein, first, by targeting P-glycoprotein (P-gp), which is one of the major cellular ATP-dependent efflux transporters for a variety of substrates, including organic anions^40^. However, when slices were incubated in aCSF containing fluorescein and verapamil (200 µM, inhibitor of P-gp^41^), fluorescein efflux was not reduced (Fig. 3A, B and D, *n*= 15 slices from 5 mice). Notably, verapamil reduced sulforhodamine B efflux in a different set of slices (Suppl. Figure 1), indicating drug activity. As a next potential mechanism of fluorescein efflux, we inhibited Oatp by probenecid (400 µM)^42^, which significantly reduced the length of fluorescein-stained vessels from 6.1 mm/mm^2^ (3.6, 8.1) to 0 (0, 0.2) (Fig. 3C and D, *p* = 1.5*10^−7^, *n* = 16 slices from 6 mice vs. 25 control slices from 9 mice, Mann-Whitney U test and Bonferroni correction). Of note, both verapamil and probenecid have also been shown to inhibit multi-drug resistance-associated proteins (Mrp)^67^, yet only probenecid inhibited fluorescein efflux. Thus, our data suggest that fluorescein efflux requires Oatp rather than P-gp or Mrp.

**Fig. 4:**
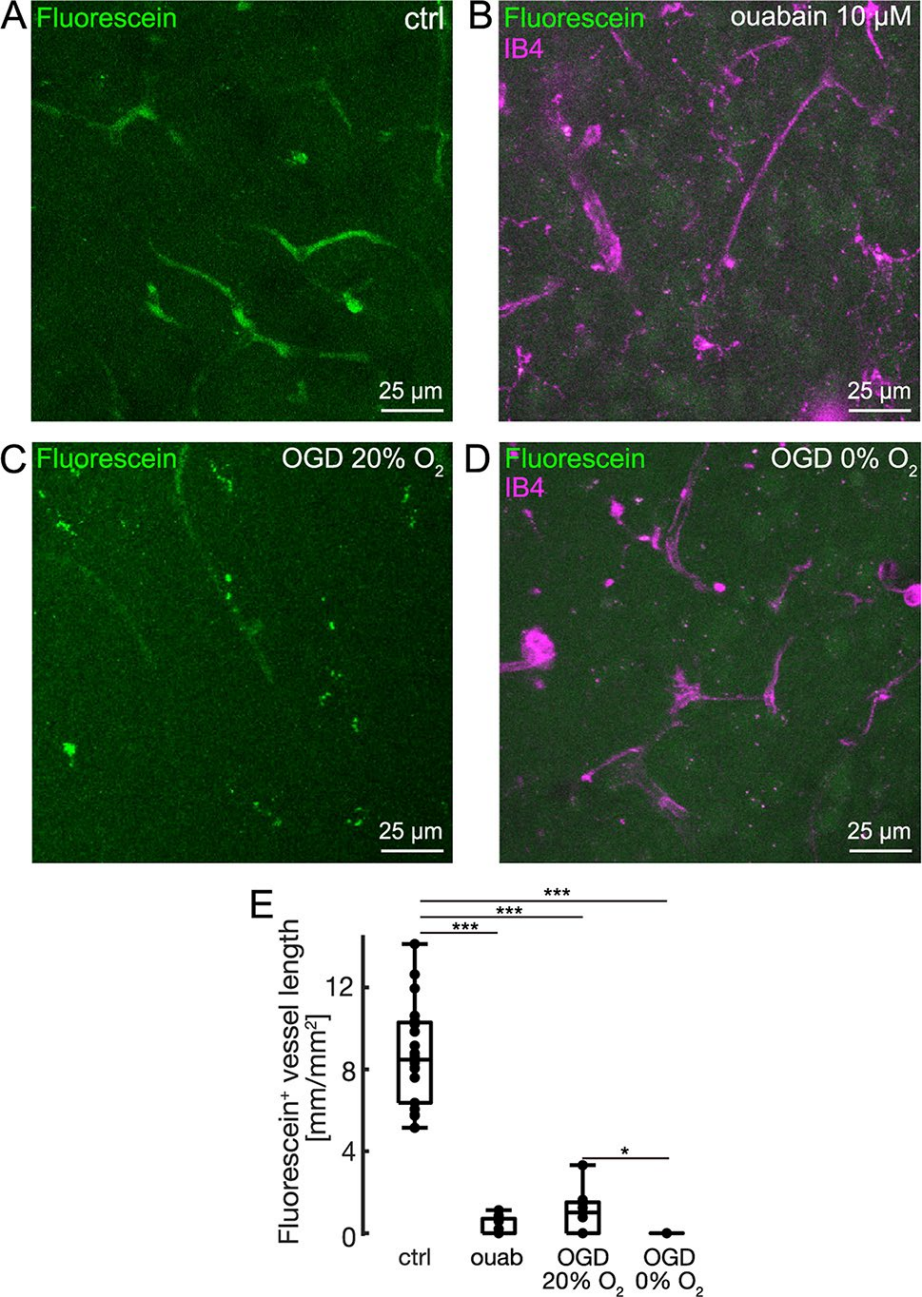
Na/K-ATPase inhibition and oxygen and glucose deprivation reduce fluorescein efflux. (**A**) Z-projection of an acute brain slice stained with fluorescein (0.5 µM; ctrl). (**B**) Z-projection as in A for a different slice co-stained with IB4 (1.45 nM) in presence of ouabain (10 µM, inhibitor of Na/K-ATPase). (**C**) Z-projection of a different brain slice co-stained with fluorescein (0.5 µM) and IB4 (1.45 nM) in oxygen- and glucose deprived (OGD) aCSF (0% O_2_, 95% N_2_, and 5% CO_2_) acquired with the same laser power as in A and B (1.5%), which is also representative for brain slices incubated with 20% O_2_, with most ROIs showing no efflux of fluorescein. Note that control slices were perfused with aCSF gassed with 95% O_2_, 5% CO_2_. (**D**) Data summary for the effect of ouabain (“ouab”, 10 µM, *n* = 11 slices from 4 mice), “OGD 20% O_2_” (*n* = 9 slices, 3 mice), and “OGD 0% O_2_” (*n*= 12 slices, 4 mice) on the length of fluorescein-positive vessels per mm^2^ compared with control slices (*n* = 22 from 8 animals, *p < 0.01, ***p < 0.0001, Mann-Whitney U test & Bonferroni post-hoc correction).

### Fluorescein efflux depends on Na/K-ATPase activity and oxidative metabolism

We previously observed fluorescein extravasation in vivo, predominantly in hypoperfused regions surrounding a primary photothrombotic ischemic lesion, following i.v. fluorescein administration in rats^43,44^. While extravasation of fluorescein was detected in all animals, little is known about the underlying transport mechanisms. To mimic the effects of critically reduced CBF, ultimately limiting ATP production required for sodium-potassium ATPase (Na/K-ATPase) activity in vitro, we incubated slices in aCSF containing ouabain (10 µM), which at this concentration primarily reduces the activity of the transporter-associated α2 and α3 isoforms of the Na/K-ATPase^45^, or in oxygen-glucose deprivation (OGD) solutions exposed to either 20% O_2_ or 0% O_2_. Inhibition of the Na/K-ATPase or OGD substantially reduced fluorescein efflux (Fig. 4A-C). Accordingly, the cumulative length of fluorescein-positive vessel fragments dropped from 8.5 mm/mm^2^ (6.4, 10.3) under control condition, i.e. carbogenated drug-free aCSF (95% O_2_, *n* = 22 slices from 8 mice), to 0.0 mm/mm^2^ (0, 0.7; *p* < 0.0001, *n* = 11 slices from 4 mice) and 1.0 mm/mm^2^ (0, 1.5; *p* < 0.0001, *n* = 9 slices from 3 mice, both Mann-Whitney U test and Bonferroni correction) in slices exposed to ouabain and OGD solution gassed with 20% O_2_, respectively (Fig. 4D). Exposing brain slices to O_2_-free OGD solution made efflux of fluorescein undetectable, as the i.v. fluorescence no longer exceeded the e.v. fluorescence (*p* < 0.0001 and *p* < 0.01 compared with control and OGD 20%, respectively, *n* = 12 slices from 4 mice, Mann-Whitney U test and Bonferroni correction; Fig. 4D). Hence, our experiments indicate active efflux of fluorescein, as i.v. fluorescence only exceeded e.v. fluorescence in the presence of oxygen and glucose as well as Na^+^/K^+^-ATPase activity.

## Discussion

We explored efflux of anionic fluorescein via its application to the brain parenchyma, which imitates blood to brain ‘leakage’, i.e. extravasation, in vitro. Efflux remains largely unnoticed after i.v. administration of markers to investigate BBB permeability due to primarily high i.v. concentrations, yet when reduced, could add to phenotypic *blood* to *brain* ‘leakage’ during BBB dysfunction. We found that parenchymal application of fluorescein indeed led to vascular accumulation indicating efflux, which was dependent on oxidative phosphorylation, Na/K-ATPase activity, and presumably mediated by Oatp. In addition, we tested other markers, which have been used to measure BBB permeability, and found efflux of another anionic low MW tracer, namely sulforhodamine B^34^, whereas we found no evidence for efflux of larger anionic FITC-conjugated dextran^24,28,46^and low MW cationic rhodamine 123^35^.

Despite the frequent use of fluorescein to detect BBB dysfunction, little is known about the underlying mechanisms for increased extravasation, and potentially reduced efflux after fluorescein extravasation. It has been shown that i.v. fluorescein does not cross a ‘healthy’ BBB or blood-retinal barrier^25,46–48^. Thus, the transfer of fluorescein from *blood* to *brain *indicates increased BBB permeability, which may result from increased paracellular transport when tight junctions break down^25^. However, parenchymal fluorescein accumulation has also been observed prior to tight junction breakdown, e.g. during the first hours after ischemic stroke in rodent models^43,44,49,50^, which indicates transcellular transfer of fluorescein from *blood* to *brain*. The transcellular mechanisms of fluorescein extravasation in the presence of increased BBB permeability remain largely unknown. One study suggested increased vesicular transcytosis following extensive somatosensory stimulation in rats^21^. Furthermore, two in vivo studies suggested that fluorescein transport may not only be directed from *blood* to *brain* but also from *brain* to *blood *by showing either an increased brain-plasma fluorescein ratio with the use of Oatp inhibitors, reducing efflux^31^, or, a decreased brain fluorescein content with upregulation of Mrp2, an anion transporter^32^. The studies relied on i.v. administration of fluorescein and were not designed to demonstrate efflux, i.e. vascular accumulation, directly. Thus, by showing fluorescein accumulation in blood vessels (i.v.> e.v fluorescence) during parenchymal application, we provide complementary evidence for efflux of fluorescein. This supports the hypothesis that analysis of fluorescein extravasation may underestimate BBB permeability due to inversely directed efflux^31,51^.

Efflux was not exclusive to fluorescein and appeared to favor low MW anionic dyes over larger or cationic molecules in the small cohort of investigated dyes. Sulforhodamine B, another low MW anionic dye, accumulated in blood vessels similarly to fluorescein, while anionic FITC-labeled dextran (3000 Da) and cationic rhodamine 123 did not. Sulforhodamine B has rarely been used as a BBB marker, yet FITC-labeled dextrans are in frequent use to measure BBB permeability and have been shown to remain on the luminal side of an intact BBB^24,25,28,52,53^. Based on our data, FITC-labeled dextran (3000 Da) is not affected by efflux and could thus be used as a marker detecting increased BBB permeability solely attributed to increased *blood* to *brain *flux. However, diffusion of molecules was shown to depend on molecular size and could on top of that be affected by the tortuosity of the extracellular space compared with homogenous media such as agarose^54,55^, ultimately affecting dye concentrations at the capillaries. Nicholson & Tao showed that compared to agarose, tortuosity in the extracellular space increases for dextrans larger than 10–40 kDa, whereas below 10 kDa diffusion would not be expected to be facilitated any more even for much smaller molecules^54^. Therefore, we do not expect a significant additional restriction for diffusion of FITC-labeled dextran (3000 Da) compared to fluorescein due to tortuosity. Taken together, as MW per se influences diffusion kinetics^54,55^, we cannot formally exclude a possible efflux by yet inactive transporters with lower affinity to FITC dextran at higher dye concentrations. Rhodamine 123 is a known substrate of P-gp^56–58^, one of the major ATP-dependent efflux transporters expressed in cerebral endothelial cells and was thus a candidate dye to study export. The lack of accumulation of rhodamine 123 in blood vessels may be due to a strictly luminal localization of P-gp^3^, which was not primarily dye-exposed, or due to abluminal efflux, when assuming expression of P-gp at the abluminal membrane as shown in rats and humans^59^. Previously, efflux by P-gp and breast cancer resistance protein was studied in isolated capillaries for N-ε-(4-nitrobenzofurazan-7-yl)-D-Lys8-cyclosporin A and BODIPY FL prazosin, respectively, demonstrating feasibility to study brain-to-blood transport in vitro^60,61^. In rodent hippocampal slice cultures, bath-applied dichlorofluorescein was shown to accumulate in blood vessels and extravasate upon seizure-induced microvascular injury, presumably via P-gp-mediated efflux followed by extravasation^62^. More recently, acute brain slices from mice and patients have been used to study BBB permeability using novel microvascular tracer injections^63^. Using this approach, Hanafy et al. showed for example that key features of the BBB, i.e. reduced trans- and paracellular transport and luminal efflux transporter activity are maintained in blood vessels in acute brain slices.

Our data suggest that efflux of sulforhodamine B was mediated by P-gp or Mrp, whereas fluorescein efflux was mediated by Oatp, as vascular accumulation of fluorescein was inhibited by probenecid (Oatp and Mrp inhibitor) but not by verapamil (P-gp and Mrp inhibitor). This is consistent with in vivo data showing increased brain fluorescein content when fluorescein was injected in presence of probenecid^31^. Remarkably, inhibition of Oatp alone, i.e. without manipulations to increase extravasation, caused increased brain fluorescein content, suggesting continuous extravasation of fluorescein across a functioning BBB counterbalanced by efflux explaining low brain fluorescein content despite extravasation. Another study showed reduced brain fluorescein content when the anion transporter ‘Mrp2’ was upregulated, again without indications for altered extravasation^32^. Taken together, these studies imply that the impermeability of the BBB to fluorescein, as previously described^46,47^, requires its export by anion transporters. However, the pathway of putative transport of fluorescein across a healthy BBB prior to efflux remains unknown and thus debatable. We show that fluorescein can be used to study efflux *in vitro.* In addition to experiments targeting transporters, the suppressed vascular accumulation of fluorescein under OGD and inhibition of the Na/K-ATPase indicate active transport. Transmembrane sodium gradients established by the Na/K-ATPase could serve as a driving force for transport and thus be the link to reduced efflux upon inhibition of the Na/K-ATPase. Consequently, reduced efflux may add to apparent fluorescein leakage from *blood* to *brain *whenever energy supply cannot meet cerebral energy demand, e.g. following stroke or cortical spreading depolarization^43,44,64^. Furthermore, reduced activity or expression of endothelial transporters should be considered when observing fluorescein extravasation in presence of intact tight junctions and no indication of increased extravasation. Interestingly, the expression of efflux transporters has become a research focus for the physiological regulation of BBB permeability during the sleep-wake cycle in drosophila and mice^20,65,66^. In mice, however, the observed circadian expression rhythms mainly affected ABC-transporters such as P-gp, but not solute carriers such as Oatp^20^. Accordingly, circadian changes were measured in brain rhodamine 123 but not in fluorescein levels. Thus, complementary evidence points to fluorescein being a substrate of Oatp rather than P-gp at the BBB.

### Limitations

(1) Experiments were exclusively based on bath application of dyes. (2) In this study, capillaries (< 5 μm) were the abundant vascular subtype. While staining of larger vessels was also observed, statistical analysis was predominantly based on capillaries (see methods). (3) We acknowledge that differences in molecular weight and charge of the dyes used in Fig. 2 may have influenced their diffusion kinetics and ultimately concentrations at the capillary wall. Therefore, although all dyes have been shown to diffuse to the imaged focal planes, we cannot exclude the possibility of efflux of rhodamine 123 and FITC dextran (3000 Da) into blood vessels at higher concentrations. (4) Probenecid and verapamil, although often used as inhibitors of Oatp and P-gp, respectively, are also inhibitors of Mrp^67^. (5) The software-assisted but largely manual quantitative analysis of blood vessel length, i.e. selection and tracing, was not free of subjectivity, which may have led to some variability. (6) Furthermore, differences in the quantified data may reflect changes in capillary density in addition to drug effects or OGD. Nevertheless, the significant results of this study stand up to variability introduced by tracing or changes in capillary density, as independent of the dye-positive vessel length, a proportion of slices were free of staining under ouabain, probenecid and OGD, whereas all control slices showed efflux of fluorescein. When applying this method to other tracers and treatments in the future, the control of capillary density could improve the sensitivity of this approach, opening up the opportunity of using this technique more widely. e.g. to characterize known or novel BBB tracers.

## Conclusions

Reduced efflux of fluorescent tracers, e.g. fluorescein, can add to phenotypic extravasation. Markers that are not subject to efflux should be used when studying pathways of *blood* to *brain* ‘leakage’. We anticipate that the combined use of different markers will help to identify specific vascular alterations in neurological disorders associated with BBB dysfunction and thus improve the underlying pathophysiological understanding, e.g. of vasogenic cerebral edema.

## Methods

Animals were housed and bred in accordance with the German Animal Welfare Act and the European Communities Council Directive (2010/63/EU) and complies with the ARRIVE guidelines. Experiments and methods were approved by the local authorities (T10/20). All experiments/methods were performed in accordance with the relevant regulations and guidelines. The study included 135 acute brain slices from 25 adult (~ 3 months-old) male mice with a C57BL/6J background (The Jackson Laboratory).

### Preparation

All experiments were conducted in acute neocortical brain slices. Following brief anesthesia by isoflurane, animals were decapitated, brains were removed and cut into coronal slices (thickness 300 µM) on a vibratome (Leica VT1200S, Leica Biosystems, Nussloch, Germany) in ice-cooled aCSF. Thereafter slices were kept in warmed aCSF (35 °C) for 30 min before being transferred to room temperature (20–22 °C).

### Solutions and drugs

Drugs were purchased from Sigma Aldrich if not stated otherwise. ACSF contained in mM: 125 NaCl, 2.5 KCl, 1.25 NaH_2_PO_4_, 26 NaHCO_3_, 1.8 MgCl_2_, 2 CaCl_2_, and 20 glucose (osmolarity ~ 305 mOsm) and was continuously gassed either with carbogen (95% O_2_, 5% CO_2_, pH 7.4), an O_2_-free gas mixture (95% N_2_, 5% CO_2_) or only exposed to room air (20% O_2_). For OGD, glucose was replaced by equimolar sucrose. Other chemicals: ouabain octahydrate (final concentration 10 µM), probenecid (400 µM), and verapamil hydrochloride (200 µM).

### Microscopy

For imaging, slices were transferred to a bath perfusion chamber (Warner Instruments, Holliston, MA, USA, perfusion rate 6–8 ml/min). To prevent movement artifacts slices were weighted with a U-shaped platinum wire holding a nylon grid. Three ROIs were selected in the somatosensory cortex (layer II-V) 20–70 μm below the slice surface. Descending arterioles were avoided on the basis of bright-field microscopy, as the capillary density is known to be lower in their vicinity (radius of ~ 50 μm)^38^. These selection criteria resulted in the presence of capillaries < 5 μm in diameter in > 99% of all ROIs, whereas vessels in the size range of 5–10 μm were present in 16–30% of all ROIs and vessels > 10 μm played a subordinate role as they were only present in 4–9% of all ROIs depending on the experimental group. Images were acquired by 2-photon laser scanning microscopy (2PLSM, excitation at 800 nm) using a Ti: Sapphire laser (Mai Tai DeepSee, Spectra-Physics, Milpitas, CA, USA) and a laser scanning microscope (Olympus Fluoview 10 M, Olympus, Tokyo, Japan) equipped with a 25x objective (XLPlan N 25x W NA:1.05, Olympus) controlled by Olympus Fluoview ASW software (version 04.01). Fluorescence was low pass filtered (690 nm) before passing a dichroic mirror (D570 nm). The two resulting channels were further bandpass filtered with 495–540 nm and 575–630 nm barrier filters (for ‘green’: fluorescein, FITC-dextran, rhodamine-123, and ‘red’ signal: IB4, sulforhodamine B, respectively). Images were acquired as xyz-stacks with a zoom factor of 2–4 (image dimensions: 512 × 512 pixels, xy-pixel-dimension 0.25–0.5 μm), covering at least 169*169 µm (x*y) and up to 20 z-steps at intervals of 1–2 μm. The stacks covered 29 μm (28.9, 35.5 μm) (median, lower and upper quartile) in the z-dimension, with no differences between the groups.

### Experimental protocols

Fluorescent dyes were dissolved in the aCSF immediately after slicing and thus continuously present in the e.v. (i.e. parenchymal) compartment of the brain slices. Alexa Fluor 568 conjugated isolectin griffonia simplicifolia-IB4 (IB4, 1.45 nM, Invitrogen) was used to label endothelium. The following dyes, which differed in molecular weight (MW) and charge were used: anionic FITC-conjugated dextran (3000 Da, 1 µM, Invitrogen), low MW fluorescein (332 Da, 0.1–0.5 µM, fluorescein sodium salt) and sulforhodamine B (559 Da, 0.1 µM), and low MW cationic rhodamine 123 (0.1 µM, 344 Da). Dye signals in the parenchyma and in vessels were detected by 2PLSM after at least 30 min of incubation, in the continuous presence of dyes in the aCSF, with dynamic wash-in experiments (Fig. 1B-C) being the only exception. The incubation period allowed fluorescein intensity to reach a plateau in the i.v. and e.v. compartment as shown in a subset of slices (Fig. 1B,C), which were not pre-incubated with fluorescein and imaged during wash-in of fluorescein (1 z-stack/min).

To investigate transport mechanisms and their dependence on oxidative metabolism, slices were exposed to dye-containing solutions in presence of drugs (see above) or OGD. Slices maintained in carbogenated aCSF containing equimolar amounts of dyes served as controls. Slices were randomly allocated to different experimental groups and were imaged in alternating order to ensure that mean dye exposure times did not differ between groups.

### Data analysis

All data were analyzed in Matlab (The MathWorks, Natick, Ma, USA). Due to their abundance (see above), capillaries were the predominant vessel subtype used for analysis. To quantify dye-positive vessel fragments, z-stacks were converted to maximum-intensity z-projections. Background was assessed by recording photomultiplier signals at employed offset and gain settings *without* laser excitation and was subtracted. No formal blinding was performed, but filenames did not allow group allocation during the analysis. Apparent dye-positive vessel fragments were traced and quantified using the function ‘drawpolyline’. Vessels were selected when they appeared brighter than neighboring e.v. space independent of a numerical threshold. Of note, isointense vessels were not visible on fluorescein staining. Retrospective analysis of slices incubated with fluorescein showed that detected vessels were at least 27% brighter than the e.v. space. Three ROIs were randomly chosen, quantified, and averaged for each slice. The corresponding mean is reported as one ‘n’ and used for statistical analysis. No vessels were excluded from analysis based on size. However, due to the rarer presence of vessels with a diameter > 10 μm in the ROIs, conclusions are more reliable for vessels of diameter < 10 μm in vitro.

### Data reporting and statistical analysis

This is an exploratory study. Due to the lack of prior evidence sample sizes could not be accurately estimated and were selected to comply with likewise research. Data are reported and shown as median and lower and upper quartiles (Q1 and Q3) unless otherwise stated. Whiskers extend to minimal and maximal values unless considered as outliers (‘+’, values > 1.5*interquartile range). Statistical inference was performed by Mann-Whitney U test and Bonferroni correction for multiple comparisons. Differences were considered significant at *p* < 0.05. Data will be made available upon reasonable request.

## Electronic supplementary material

Below is the link to the electronic supplementary material.

Supplementary Material 1

## Data Availability

Data will be made available by the corresponding author upon reasonable request.
