# Supplementary material for: Brain-to-blood transport of fluorescein in vitro
Source: Sci Rep. 2024 Oct 26;14:25572. doi: 10.1038/s41598-024-77040-2 (PMC11513102; doi:10.1038/s41598-024-77040-2)
Supplement: Supplementary file 1 — Supplementary Material 1 [file 41598_2024_77040_MOESM1_ESM.pdf]

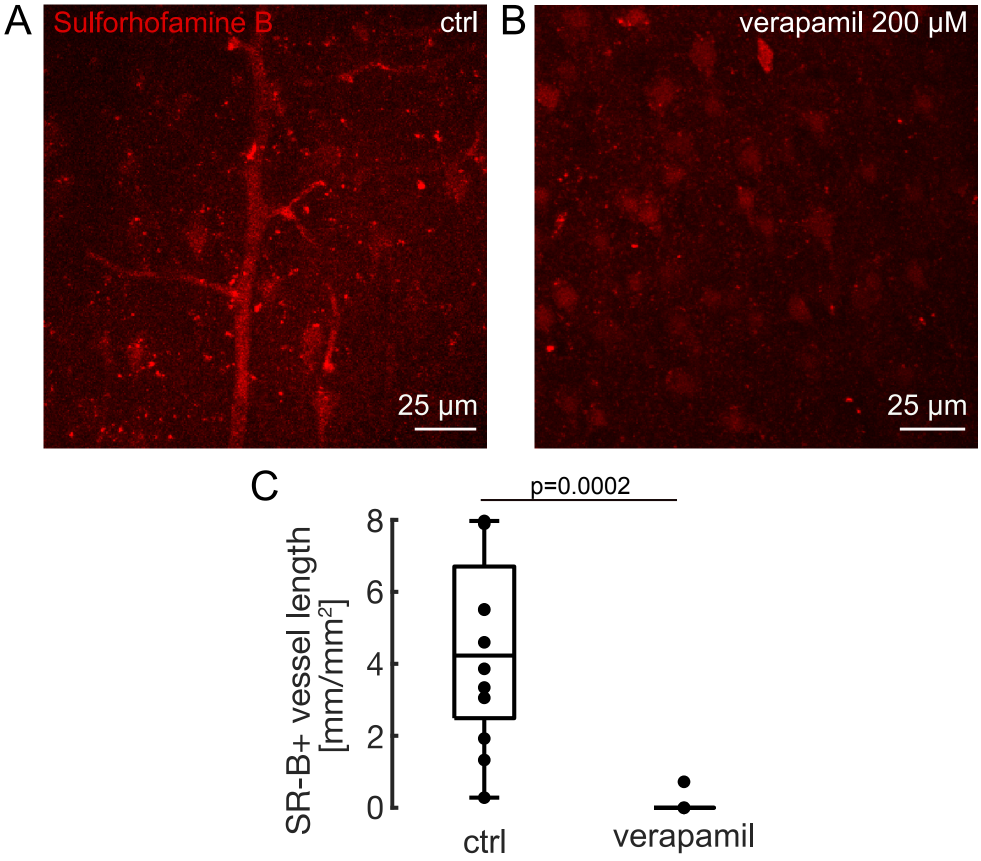

Supplementary Fig. 1 Verapamil inhibits efflux of sulforhodamine B

A) Z-projection of acute brain slice incubated with sulforhodamine B (0.1 µM; ctrl).

B) Z-projection as in A for a different slice incubated with sulforhodamine B (0.1 µM) in presence of verapamil (200 µM).

C) Boxplot summarizing the effect of verapamil (n=8 slices, 3 mice) compared to untreated control slices (n=12 slice, 4 mice, Mann-Whitney U test).
